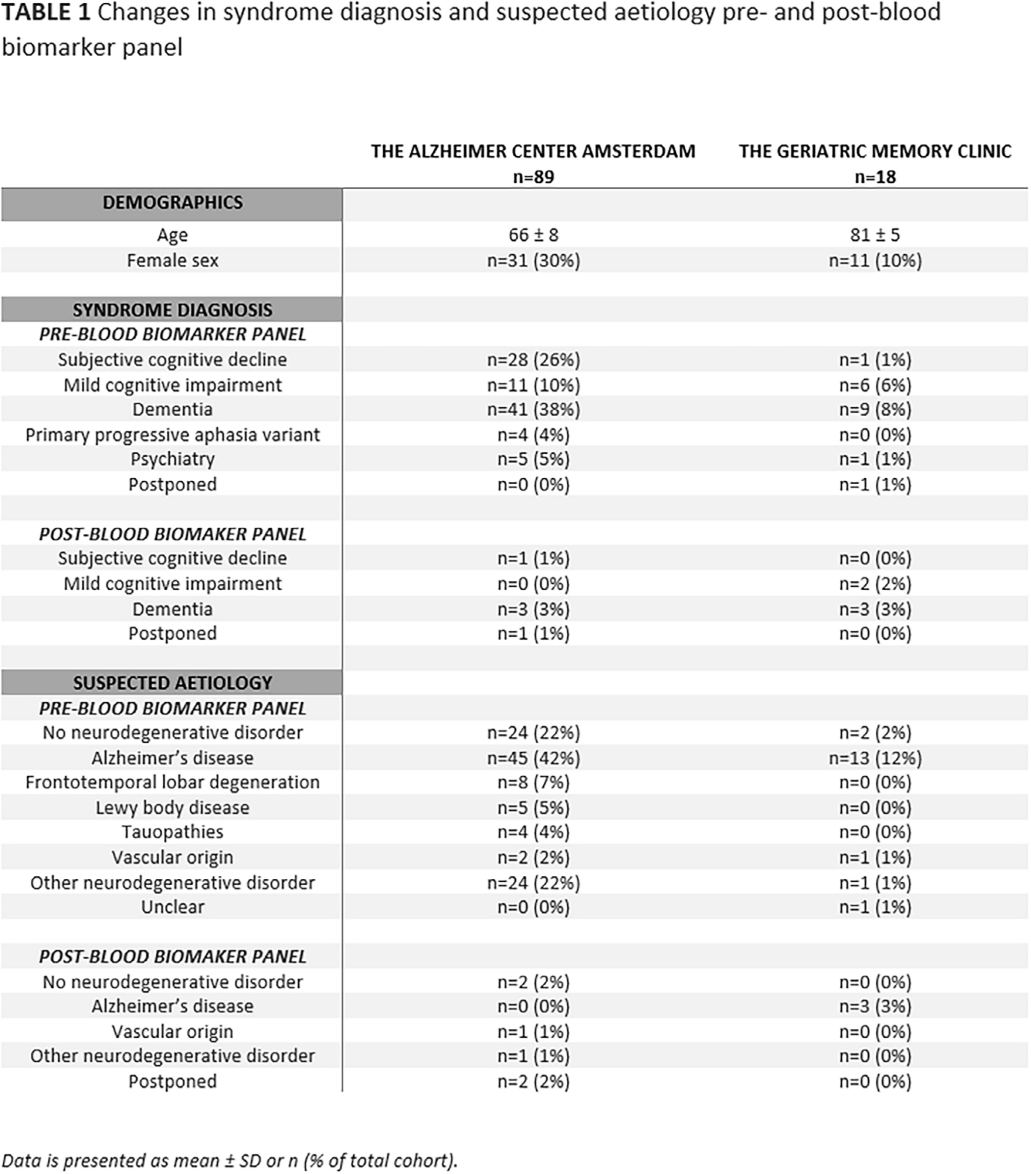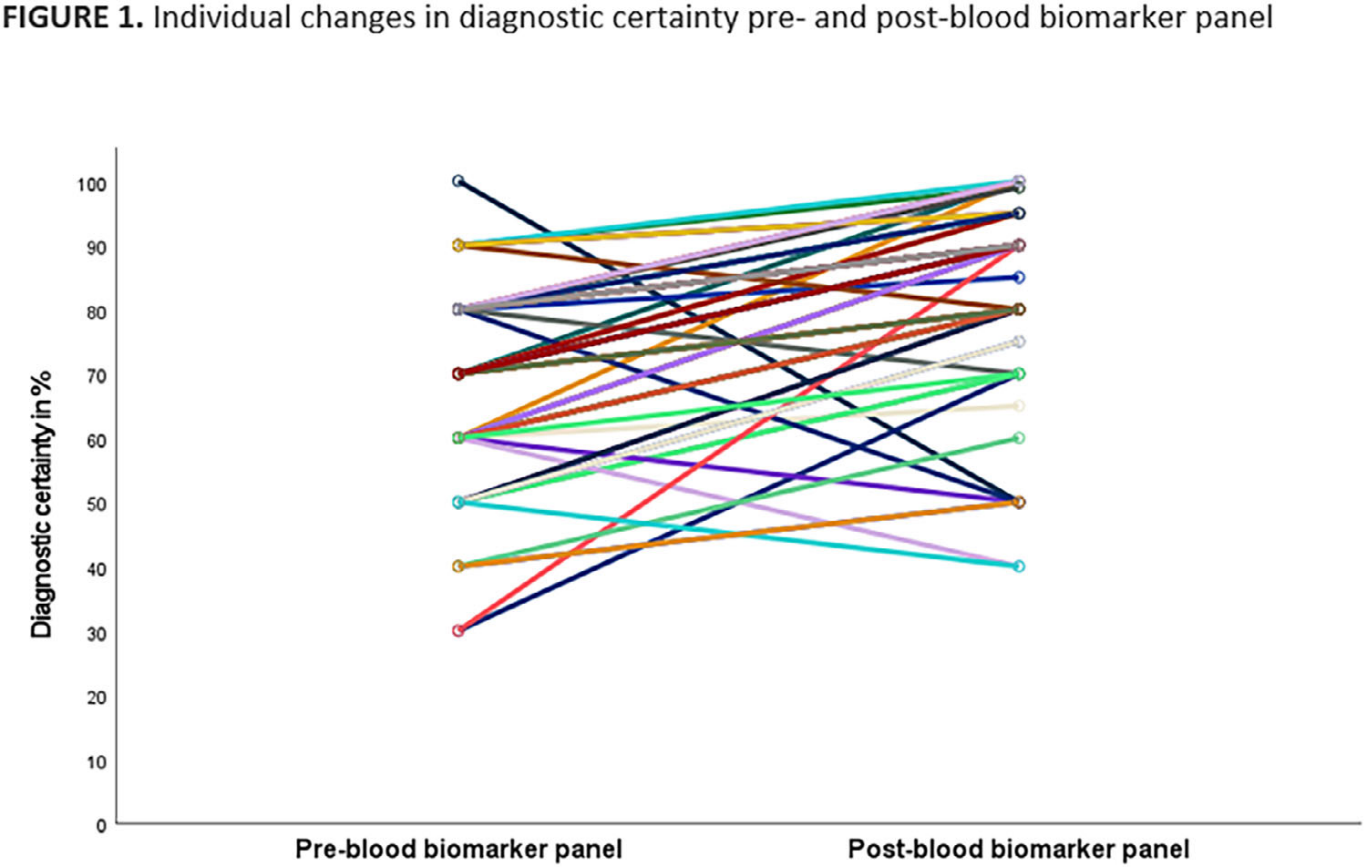# Clinical consequences of the Alzheimer’s blood‐based biomarker panel: a prospective evaluation in memory clinics ‐ the CANTATE project

**DOI:** 10.1002/alz.091259

**Published:** 2025-01-09

**Authors:** Sinthujah Vigneswaran, Inge M.W. Verberk, Lynn Boonkamp, Rebecca Z. Rousset, Thomas Claessen, Marissa D. Zwan, Rik Ossenkoppele, Majon Muller, David H Wilson, Afina Willemina Lemstra, Yolande A.L. Pijnenburg, Wiesje M. van der Flier, Charlotte Teunissen, Argonde C. van Harten

**Affiliations:** ^1^ Alzheimer Center Amsterdam, Neurology, Vrije Universiteit Amsterdam, Amsterdam UMC location VUmc, Amsterdam Netherlands; ^2^ Neurochemistry Laboratory, Department of Clinical Chemistry, Amsterdam Neuroscience, Vrije Universiteit Amsterdam, Amsterdam UMC, Amsterdam Netherlands; ^3^ Amsterdam Neuroscience, Neurodegeneration, Amsterdam Netherlands; ^4^ Lund University, Clinical Memory Research Unit, Lund Sweden; ^5^ Department of Internal Medicine, Geriatric Medicine Section, Amsterdam Cardiovascular Sciences Institute, Vrije Universiteit Amsterdam, Amsterdam UMC, Amsterdam Netherlands; ^6^ Quanterix, billerica, MA USA; ^7^ Alzheimer Center Amsterdam, Neurology, Vrije Universiteit Amsterdam, Amsterdam UMC, Amsterdam Netherlands

## Abstract

**Background:**

Blood‐based biomarkers (BBM) are emerging as minimally invasive, scalable and relatively low‐cost options for discriminating different neurodegenerative diseases. Before implementation in clinical practice can take place, it is important to determine their real‐world clinical validity in patients presenting at a memory clinic. Therefore, we prospectively evaluated changes in diagnosis and diagnostic confidence resulting from the use of a BBM panel tailored to common differential diagnostic considerations (Verberk et al., Mastermind of the Alzheimer’s blood‐based biomarkers: development of cut‐offs and a visualization tool for use in clinical practice. https://doi.org/10.1002/alz.078748).

**Methods:**

Consecutive patients who visited the Alzheimer Center Amsterdam or the Center Of Geriatrics Amsterdam (COGA) for a standardized diagnostic workup between September‐December 2023 and provided informed consent were included. Diagnoses were determined at a multidisciplinary meeting. BBM panel with pre‐specified cut‐off values (plasma pTau181, GFAP and NfL, Quanterix Corporation, USA) were disclosed after the clinical work‐up, neuropsychological tests and MRI results were presented on a weekly basis reflecting real world clinical practice. We evaluated syndrome diagnosis, suspected aetiology and confidence in the etiological diagnosis before and after disclosing BBM results.

**Results:**

A total of 107 patients with a mean age of 68 ± 9 years and 42 females (39%) were included. The blood biomarker panel led to change in syndrome diagnoses in 10 patients (9%) and change in suspected aetiology in 9 patients (8%) (Table 1). Among those for whom both syndrome diagnosis and suspected aetiology remained unchanged (n=92, 86%), diagnostic certainty changed in 89 patients (83%) after showing the BBM panel. In these patients, the diagnostic confidence changed from 70% [IQR = 60 ‐ 80] to 90% [IQR = 80 ‐ 90]; P < .001) after showing the BBM panel results (Figure 1). The diagnostic confidence increased in 69 (64%) patients and decreased in 20 (19%) patients.

**Conclusion:**

A BBM panel specific to individual differential diagnostic considerations generally led to an increase in diagnostic confidence and rarely resulted in a different diagnosis. These results suggest that BBM have added value in daily clinical practice. This is an ongoing study with inclusion rate of 15 persons per week, updated results will be presented at AAIC2024.